# Guides and cheats: producer–scrounger dynamics in the human–honeyguide mutualism

**DOI:** 10.1098/rspb.2023.2024

**Published:** 2023-11-08

**Authors:** Dominic L. Cram, David J. Lloyd-Jones, Jessica E. M. van der Wal, Jess Lund, Iahaia O. Buanachique, Musaji Muamedi, Carvalho I. Nanguar, Antonio Ngovene, Shirley Raveh, Winnie Boner, Claire N. Spottiswoode

**Affiliations:** ^1^ Department of Zoology, University of Cambridge, Cambridge, Cambridgeshire CB2 3EJ, UK; ^2^ FitzPatrick Institute of African Ornithology, University of Cape Town, Rondebosch 7701, South Africa; ^3^ Mbamba village, Niassa Special Reserve, Mozambique; ^4^ EO Wilson Biodiversity Laboratory, Gorongosa National Park, Mozambique; ^5^ School of Biodiversity, One Health and Veterinary Medicine, College of Medical, Veterinary and Life Sciences, University of Glasgow, Graham Kerr Building, Glasgow G12 8QQ, UK

**Keywords:** producer–scrounger, mutualism, foraging strategies, human–wildlife cooperation, *Indicator indicator*, greater honeyguide

## Abstract

Foraging animals commonly choose whether to find new food (as ‘producers’) or scavenge from others (as ‘scroungers’), and this decision has ecological and evolutionary consequences. Understanding these tactic decisions is particularly vital for naturally occurring producer–scrounger systems of economic importance, because they determine the system's productivity and resilience. Here, we investigate how individuals' traits predict tactic decisions, and the consistency and pay-offs of these decisions, in the remarkable mutualism between humans (*Homo sapiens*) and greater honeyguides (*Indicator indicator*). Honeyguides can either guide people to bees’ nests and eat the resulting beeswax (producing), or scavenge beeswax (scrounging). Our results suggest that honeyguides flexibly switched tactics, and that guiding yielded greater access to the beeswax. Birds with longer tarsi scrounged more, perhaps because they are more competitive. The lightest females rarely guided, possibly to avoid aggression, or because genetic matrilines may affect female body mass and behaviour in this species. Overall, aspects of this producer–scrounger system probably increase the productivity and resilience of the associated human–honeyguide mutualism, because the pay-offs incentivize producing, and tactic-switching increases the pool of potential producers. Broadly, our findings suggest that even where tactic-switching is prevalent and producing yields greater pay-offs, certain phenotypes may be predisposed to one tactic.

## 1. Introduction

Foraging animals can invest time and energy into finding new food resources (the ‘producer’ tactic), or search for opportunities to eat food found by others (the ‘scrounger’ tactic), and this decision can have substantial ecological, evolutionary, and economic consequences [1–3]. Early work on producer–scrounger dynamics established that population-level rates of producing and scrounging are frequency-dependent, and that the stable equilibrium is influenced by characteristics of the population and the resource at stake [4,5]. However, less attention was given to the individuals within these populations and the consequences of their variable, complex phenotypes on decisions to produce or scrounge [6]. This focus on populations rather than individuals left it unclear whether individuals show fidelity to a single tactic or flexibly switch, and whether phenotypic traits predispose certain individuals to either tactic. The extent of switching and the degree of phenotypic matching to a given tactic are likely to determine the distribution of resources across individuals, the opportunities for new individuals to produce or scrounge, and the resilience of the overall system to perturbation.

To understand whether and why individuals are faithful or flexible in their tactic decisions, studies must observe individuals repeatedly under varying conditions, and consider their states and phenotypic traits that are likely to influence these decisions. Recent studies revealed that, first, consistent individual differences mean that behavioural plasticity can be far more limited than assumed in some theoretical models [7]. As a result, an individual may be unable to adaptively switch tactics in response to changes in conditions [8–10]. Second, phenotypic differences can predispose some individuals to one tactic, and ongoing work is attempting to identify generalized associations between traits and producing or scrounging. In some systems, larger or more dominant individuals tend to scrounge, because they are better able to displace others at the food resource [11–13]. In other systems, dominants produce more, perhaps because this tactic is high-reward but costly or impossible for subordinates to do [14,15]. The role of individual condition in shaping tactic choice is also variable, with individuals in poor condition being more likely to scrounge in some systems [12,16] or to produce in others [17–19], and further work is clearly needed. Much of the empirical work to date involves controlled conditions in which novel producer–scrounger games are generated through experimental feeding (e.g. [10,20–22]; but see [13,23,24]). These experiments elegantly test the predictions of theoretical models, but their simulated foraging scenarios do not necessarily reflect producer–scrounger dynamics in nature. Wild individuals have evolved adaptive responses to naturally occurring producer–scrounger games, and understanding these responses therefore relies on data collected in the wild under natural conditions.

Clarifying the determinants and consistency of tactic choice is especially important in producer–scrounger systems of economic or cultural significance, because the system's overall productivity (and therefore its economic value) and its stability are dictated by the number of individuals that produce and how often they do so [3]. Flexible tactic-switching could bolster these systems' productivity and robustness to perturbation, by allowing *all* individuals to be potential producers and facilitating within-generation increases in the number of individuals producing. Producer activity is also strongly affected by how much of a food source they are able to monopolize before scroungers arrive (the ‘finder's advantage’ [7,24,25]), and quantifying tactic pay-offs at a given patch is therefore a key step in understanding productivity and resilience. For example, if the finder's advantage is minimal, tactic-switching could instead allow individuals to abandon producing altogether, leading to a productivity crash. Remarkably, relatively little is known about the resilience and productivity of naturally occurring producer–scrounger systems, and the factors that might safeguard them or lead to their collapse [26].

Here, we investigate the repeatability, the phenotypic correlates, and the consequences of producing and scrounging for greater honeyguide birds (*Indicator indicator*, hereafter ‘honeyguides’) that engage in a facultative foraging mutualism with humans (*Homo sapiens*). In this rare example of human–wildlife cooperation [27], a honeyguide leads the human to the location of a bees' nest, using vocal signals and visual cues [28–30]. The human then harvests the honey, and the producer honeyguide (that invested time and effort in locating the bees’ nest and energetically guiding the human to it) is able to supplement its insectivorous diet with beeswax. Without the human breaking open the bees' nest and subduing the bees, honeyguides have extremely limited access to beeswax [28]. During the honey-hunt and subsequent harvest, the behaviour of both the human and honeyguide are conspicuous (typically involving reciprocal calling, the felling or opening of a tree trunk with an axe, and the use of fire and smoke to subdue the bees [30,31]), and the resulting resource can be large and difficult to monopolize. Consequently, scrounging honeyguides arrive after the human has left and scavenge on beeswax without having invested in locating the bees’ nest and guiding the human [32]. The productivity of the human–honeyguide mutualism, which has significant ecological, economic and cultural importance [29,30,32–34] is therefore strongly influenced by the outcome of the producer–scrounger game among honeyguides. It is not known whether aspects of this game are likely to strengthen the mutualism, or make it vulnerable to human cultural change or prevalent ‘cheating’ by scroungers. Human–honeyguide cooperation has already declined in many parts of Africa, and several other cases of human–wildlife cooperation have gone extinct in the last two centuries [34], leaving the future of this likely ancient cooperative foraging partnership uncertain.

Specifically, we used the human–honeyguide mutualism to address three gaps in our understanding of producer–scrounger dynamics. First, we estimated individual consistency in the propensity to guide among wild honeyguides. Assessing how faithful or flexible tactic decisions are under natural conditions is crucial for understanding whether the population rate of guiding arises through tactic-switching by all individuals, or consistent producing by a minority. The abundant opportunities to scavenge beeswax following honey-hunts at our study site (including those in which the honey-hunter located the bees' nest without the help of a honeyguide) lead us to expect that tactic-switching will be common [32].

Second, we investigated which traits are associated with guiding and scrounging, to determine whether certain phenotypes are predisposed to either tactic. The hypothesis that more competitive individuals are typically better able to displace rivals in order to scrounge (e.g. [11]) predicts that guiding will be most prevalent in honeyguides that are either small or in poor condition. Alternatively, it has been suggested that only the most competitive individuals are able to produce [12,16], and this hypothesis would predict that guiding will be limited to larger, heavier honeyguides or those in the best condition. We tested the predictions of these hypotheses by relating guiding propensity to a suite of individual traits, including body mass, tarsus length, sex and two markers of individual condition: plumage quality and relative telomere length (an integrative marker of somatic state [35]). We also examined associations between guiding propensity and age. Previous work indicates that tactic choice can be influenced by prior experience [36–39], and older birds may therefore guide more frequently if learning improves their ability to do so (beyond the minimum age of individuals in this study of approx. 1 year).

Third, we quantified the pay-offs of producing and scrounging, in terms of access to beeswax at a given site. Almost all theoretical models of producer–scrounger dynamics assume that the individual that produces a food source will consume more of it than any individual(s) scrounging from that source, but this core assumption has rarely been tested beyond laboratory-controlled conditions [7]. The extent of the finder's advantage at a given site (relative to the pay-offs of scrounging at that site) is also likely to affect the stability and productivity of the system. Given that guiding behaviour (the producer tactic) thrives at our study site [30], we expect that guiding individuals will have substantially better opportunities to feed on the beeswax.

## 2. Methods

### (a) Study site and field methods

We carried out this study within the Niassa Special Reserve (L5 Concession) in northern Mozambique [30,32]. The study area is visited by Yao honey-hunters who live in Mbamba village, which has a population of *ca.* 2000 including at least 20 professional honey-hunters [32], including I.O.B., M.M. and C.I.N. Observations of honeyguides' producing and scrounging behaviour took place during 9–29 October 2018, 30 August–5 October 2019 and 3–19 September 2022; population data underpinning our analyses were also collected in 2013–2018.

Our data collection took place in four stages. First, a group comprising between one and three honey-hunters and between one and four observers (total group size range 2–5, 3.32 ± 0.85, mean ± s.d.), walked within our study area seeking a guiding honeyguide. One of the honey-hunters was randomly designated as the caller each day, and carried a Garmin eTrex 30 GPS unit (Garmin, USA) which recorded their track. The caller gave the recruitment call inviting a honeyguide to cooperate (‘brrrr-hm’ [30]). Second, when a honeyguide began to guide the calling honey-hunter by giving the specific chattering guiding call, an observer confirmed its identity by photographing its unique colour-ring combination using a Nikon D750 or D7500 dSLR camera (Nikon, Japan) with a 300 mm lens and a 1.4× teleconverter. In seven cases, the guiding bird was not ringed but its identity could be confirmed through unique breakages in its feathers (which were visible in photos while it guided, and confirmed when the bird was captured within 24 h). Any ringed honeyguides that were seen but did not give the guiding call were also photographed, to record their lack of guiding behaviour. Third, the calling honey-hunter followed the guiding bird to the bees' nest, and harvested the honey in their usual manner, using smoke to subdue the bees and an axe to enlarge the nest cavity, often reached by felling the tree. Fourth, after the honey had been harvested, we captured honeyguides using clap-net traps (Moudry Traps, Říčany, Czech Republic) baited with beeswax (and, rarely, beeswax placed underneath mist-nets). No other beeswax was accessible to the honeyguides during trapping sessions. Any unringed individuals were fitted with a metal ring with an ID number, and a unique combination of two colour rings for field identification.

The above protocol generated three datasets. First, we produced a dataset of repeated observations of guiding and scrounging behaviour linked to individually recognizable honeyguides. Each time an identifiable honeyguide attempted to guide the honey-hunter (by giving the distinctive guiding call [30]), we recorded this as a guiding encounter along with its ID number. Other encounters were considered scrounging encounters and involved (i) honeyguides approaching the calling honey-hunter without giving the guiding call, (ii) honeyguides approaching during the harvest of the bees’ nest (i.e. likely searching for scrounging opportunities), and (iii) honeyguides that were captured at a bees' nest to which they did not guide (i.e. actively scrounging).

Second, our captures yielded information about individual honeyguides’ phenotypic traits. For each capture, we measured body mass (using a spring scale accurate to 0.5 g, Pesola Präzisionswaagen, Switzerland), tarsus length (using callipers accurate to 0.01 mm, Mitutoyo, Japan), and counted the number of fault bars on the honeyguide's primary, secondary, and tail feathers. Fault bars are malformations caused by adversity suffered during their growth, and can therefore provide a record of the stress an individual experienced during their most recent moult [40]. We also recorded the honeyguide's sex and age class (juveniles have yellow plumage up to age approx. 1 year, which adults lack [41]). We calculated a continuous variable termed ‘minimum age’ by estimating each individual's latest possible hatching date, according to whether they were a juvenile or adult at first capture. For individuals captured as juveniles or immatures, this was estimated to be 1 year prior to the first capture, and for those first captured as adults, it was estimated to be 2 years before the first capture. For a subset of 31 birds, we collected a small blood sample for the measurement of relative telomere lengths in whole blood (comprising primarily erythrocytes, with white blood cells and other cell types, hereafter ‘relative telomere length’). We collected 40 µl of whole blood in a heparinized capillary tube by puncturing the brachial vein using a 26-gauge needle. Within 3 min, this blood was ejected into an Eppendorf containing 1 ml of 95% ethanol. Full details of telomere length estimation are available in the electronic supplementary materials. Briefly, we used the qPCR method [42] with an Mx3005P qPCR system (Agilent Technologies) to determine the ratio of the telomere repeat copy number to that of a single copy or non-variant control gene, and we calculated these values using the method developed by Pfaffl [43].

Third, our capture protocol allowed us to record the order in which honeyguides attempted to feed on the beeswax. We could thus determine whether the first individual to approach and feed on the beeswax reward provided by the honey-hunters was the individual that guided to that bees' nest, or a scrounging individual. Honeyguides show a strong preference for the purest beeswax (which is white or pale yellow and not associated with pollen, honey or larvae), and the first honeyguide to arrive at the beeswax feeds on this highly prized beeswax or flies away with it [32,44,45]. As such, arriving first at the resource provides the best access to the most valuable beeswax.

### (b) Statistical analysis

Statistical analyses were carried out in R (version 4.2.2, [46]) unless otherwise specified. The homoscedasticity and normality of residuals were inspected visually and all continuous predictors were scaled to a mean of zero and standard deviation of one.

#### (i) Is the propensity to guide or scrounge repeatable within individuals?

We extracted a dataset including all encounters with identifiable honeyguides, and whether that individual guided or scrounged on each encounter (*n* = 180 encounters of 91 individuals, range = 1–7 encounters per individual, mean ± s.d. = 1.98 ± 1.28). We used the *rptR* package (version 0.9.22) [47] to calculate the observed repeatability (*R*_obs_) for our dataset, using a model formula with guiding or scrounging as the response, and ID number as the only random term. To test whether this was significantly higher than we would expect under random chance, we randomized the dataset by shuffling the individual IDs, and calculated *R*_perm_ values across 1000 such randomized permutations (results were qualitatively identical if further permutations were run). We then statistically compared *R*_obs_ against the *R*_perm_ distribution.

#### (ii) What factors are associated with variation in propensity to guide?

We used a full model approach to test whether our predictors of interest were significantly correlated with the propensity to guide or scrounge. The significance of all terms was estimated by removing them from the full model using a likelihood-ratio test for model comparison and an alpha of 0.05. The model response was a binomial term comprising the number of times an individual guided the honey-hunter, and the number of times it scrounged, within a given field season. We included the individual's sex as a factor, and their minimum age, body mass, tarsus length and the number of feather fault bars as covariates. To test whether body mass predicted guiding propensity differently in males and females, we included the two-way interaction between sex and body mass. The field season was included as the only random term. The dataset included 91 observations across three field seasons. Given that only six birds were seen in multiple seasons (and therefore represented more than a single row in the dataset), we did not include a random term for individual ID. Results were qualitatively identical if we did so. Models were constructed using the *lme4* package (version 1.1–31) [48], with a binomial error distribution. Pearson's correlation tests indicated a significant negative association between minimum age and body mass (*p* = 0.029, estimate: −0.23) and a significant positive association between tarsus length and body mass (*p* < 0.001, estimate: 0.68). However, there was substantial unexplained variation despite these positive associations (see electronic supplementary materials) and the variance inflation factor (VIF) of all models was less than four, suggesting low to moderate multicollinearity that does not require statistical adjustment [49]. All other correlations between covariates were non-significant.

For a subset of 31 individuals for which we had blood samples, we tested whether the propensity to guide or scrounge was predicted by relative telomere lengths. We created a second binomial linear mixed effects model with the same response term, and included relative telomere length as the only covariate and field season as the random term. We were unable to include other predictors due to the limited sample size of this dataset.

#### (iii) How does guiding and scrounging affect access to the beeswax reward?

We hypothesized that a major benefit of guiding is being the first individual to arrive at the beeswax after the honey-hunter has harvested the nest. To test this, we calculated the frequency with which the first individual to arrive at a beeswax harvest was the individual that guided the honey-hunter to the bees' nest yielding the harvest. We identified the guiding bird on 23 separate honey-hunts, and subsequently captured any honeyguides feeding on the resulting beeswax (as outlined above). For each harvest site, we scored whether the first bird to be captured was the guiding bird.

To test whether the observed frequency with which the guiding bird arrived first at the beeswax differed from chance, we generated a distribution of expected frequencies under random chance. This depends on the total number of other honeyguides present at the harvest site (e.g. if 10 individuals are present, the random probability would be 10%). To calculate a distribution of expected probabilities that the guiding bird would arrive first, we estimated how many birds would be present at harvest sites following honey-hunts. Full details of these estimates are included in the electronic supplementary materials. Briefly, we first estimated the density of honeyguides at our field site, using a capture–mark–recapture analysis in the Program MARK (version 9.0) [50] over the period 2013–2019. Then, we calculated the area exposed to honey-hunters’ ‘brrrr-hm’ vocalizations on 30 honey-hunts, by combining GPS tracks with an estimate of the distance over which the calls are audible. We combined these areas with our estimate of the honeyguide density (16.58 honeyguides per km^2^), to calculate how many honeyguides would likely have heard the calling honey-hunter and therefore had the potential to arrive first at the beeswax (mean ± s.d.: 23.7 ± 12.1 individuals, range 6.3–57.6). This estimate is likely to be conservative as it does not include honeyguides attracted by the sound of the honey-hunter's axe chopping the tree trunk, or the tree falling, or the sight or smell of the smoke used to subdue the bees. We generated a distribution of expected probabilities that the guiding honeyguide would arrive at the beeswax first by taking the reciprocal of our distribution of the estimated number of honeyguides that heard the honey-hunters' calls (e.g. when a honey-hunter was estimated to have been heard by 20 honeyguides, the expected probability that the guide would arrive first was 1/20). Using this probability distribution, we permuted 100 000 randomizations in which, for each of the 30 honey-hunts, the guiding honeyguide did or did not arrive first according to the probability distribution, and statistically contrasted this expected distribution with our observed value using a permutation test.

## 3. Results

### (a) Is the propensity to guide or scrounge repeatable within individuals?

**Figure 1.**
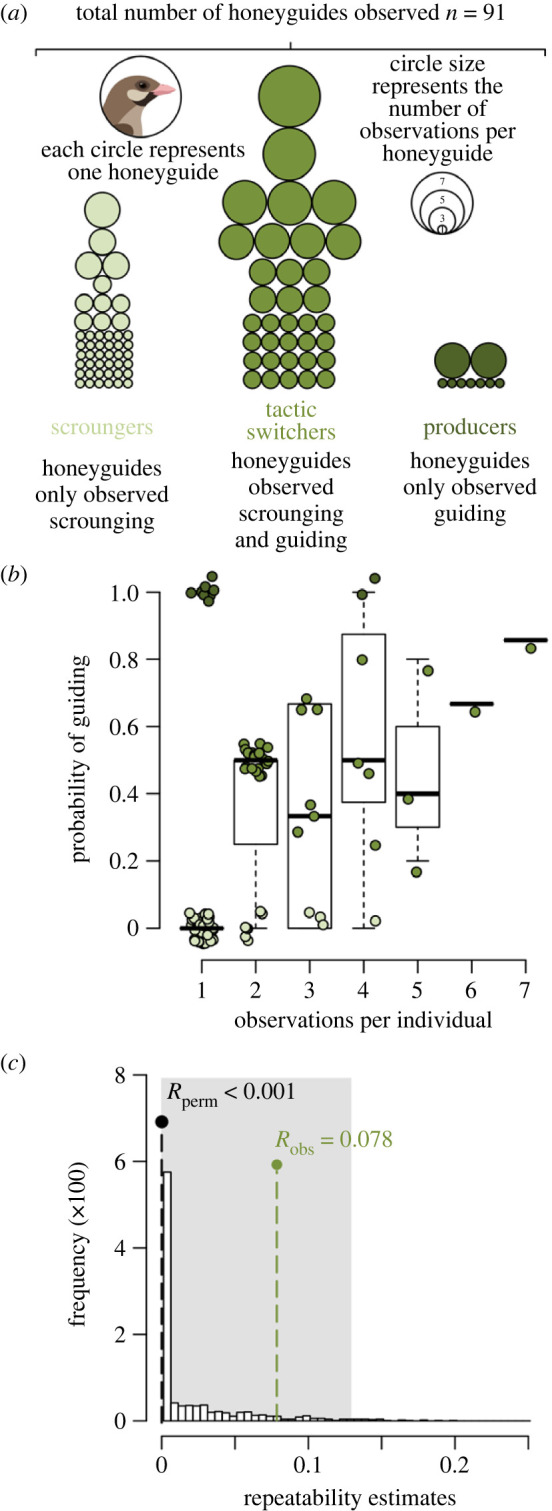
Rates of guiding and scrounging in wild honeyguides. (*a*) A graphic illustrating how many birds consistently scrounged (light green), switched tactics (mid green) or consistently guided (dark green). Each circle is an individual honeyguide, and circle sizes indicate the number of observations for each of the 91 individuals. (*b*) A boxplot showing how the number of observations per individual related to the probability each individual guided. Overlaid points show raw data, jittered on the *x*- and *y*-axes to avoid obscured overlaps, and coloured to match the three tactic categories in panel (*a*). (*c*) Frequency histogram of the repeatability estimates from 1000 randomized permutations of the data. The median of these *R*_perm_ values was zero (black dotted line). The repeatability estimate *R*_obs_ for the observations of guiding and scrounging behaviour was 0.078 (green dotted line). *R*_obs_ fell within the 95% confidence intervals of the permutations (grey shaded area), indicating that repeatability in guiding and scrounging behaviour is low. Honeyguide illustration credit: Jess Lund.

Overall, 69 of 180 (38%) honeyguide encounters were guiding rather than scrounging, demonstrating that there are fewer opportunities to produce than there are to scrounge in this system. Of the 91 individuals in our dataset, 47 only scrounged (51.6%), nine only produced (9.9%) and 35 employed both tactics (38.5%, figure 1*a*). The mean individual probability of guiding for a given individual was 29.6% (figure 1*b*).

Our analysis of repeated observations of guiding and scrounging behaviour in honeyguides suggests that their individual consistency is low and there is widespread tactic-switching. The observed repeatability estimate *R*_obs_ was 0.078 (where values of 0 and 1 indicate no repeatability and complete repeatability, respectively). This estimate fell within the 95% confidence intervals of the repeatability estimates drawn from our randomized permutations (*R*_perm_, figure 1*c*, 95% confidence intervals: 0–0.12). *R*_obs_ was greater than 88% of the *R*_perm_ values, and was marginally non-significant (*p* = 0.07). Together, these results suggest that there is likely low, but detectable, repeatability in guiding and scrounging behaviour.

### (b) What factors are associated with variation in propensity to guide?

**Figure 2.**
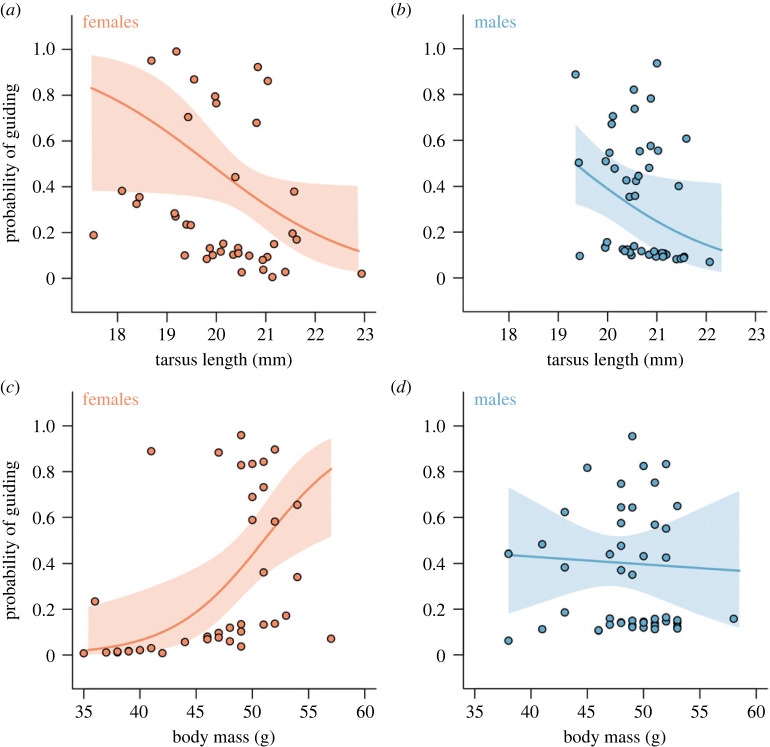
Predictors of a honeyguide's probability of guiding rather than scrounging. (*a*) and (*b*) In both female (orange) and male (blue) honeyguides, individuals with a longer tarsus were significantly less likely to guide. (*c*) In female honeyguides, body mass positively predicted the propensity to guide. (*d*) By contrast in males, body mass was not associated with guiding propensity, such that there was a significant sex × body mass interaction. In all panels, lines show the predictions from the full model, with all other covariates held at their mean value. Shaded polygons indicate the 95% confidence intervals of these predictions. Points indicate the full model's partial residuals for each prediction (i.e. the residuals after subtracting the contributions of all other explanatory variables, [51], extracted using the *ggeffects* package (version 1.3.0) [52]. In (*a*) and (*b*) points are jittered on the *x*-axis to avoid obscured overlaps.

We investigated whether the propensity to guide or scrounge was predicted by individual traits. We found that honeyguides with the longest tarsi very rarely guided (figure 2*a,b*; electronic supplementary material, table S1, χ_1_^2^ = 4.22, *p* = 0.04, *n* = 88 observations). Controlling for this significant tarsus effect, we found that guiding propensity was also predicted by the interaction of body mass and sex (figure 2*c,d*; $\chi_{1}^{2}=8.29$, *p* = 0.004). In males, variation in body mass was not associated with the propensity to guide or scrounge, but in females there was a strong positive association, such that the lightest females guided in fewer than 3% of encounters, compared to 82% in the heaviest. Guiding propensity was not significantly predicted by minimum age or the number of feather fault bars (both $\chi_{1}^{2}<2.01$, *p* > 0.16). The model's marginal pseudo *R*^2^ was 24.5% [53].

For a subset of our data, we measured the honeyguides' relative telomere lengths. In a model restricted to this dataset, relative telomere length was not significantly associated with propensity to guide (electronic supplementary material, table S2, $\chi_{1}^{2}=0.22$, *p* = 0.64, *n* = 31 individuals).

### (c) How does guiding and scrounging affect access to the beeswax reward?

**Figure 3.**
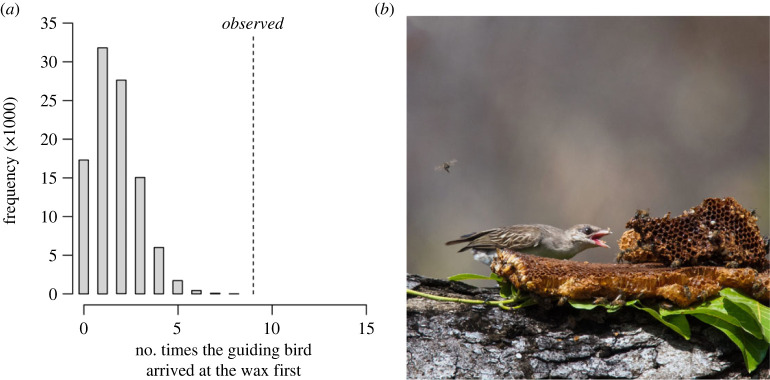
(*a*) The observed frequency that the guiding bird arrived at the wax first (9/23 or 39% of guided honey-hunts and resulting harvest sites, dotted line). This observed frequency was significantly greater than the expected frequencies under random chance (grey histogram bars). The grey bars show a histogram of the frequency that the guiding bird arrived first under random chance, drawn from 100 000 permutations. The randomized probability that the guide would arrive first in each permutation was calculated by estimating the number of honeyguides present at 30 real honey-hunt harvests, and calculating the reciprocal for each harvest. (*b*) A honeyguide feeding on a pile of beeswax left by a honey-hunter from the Yao community in Niassa Special Reserve, northern Mozambique. Honeyguide image credit: Dominic Cram.

Among 23 honey-hunts in which the guiding bird's identity was confirmed, and the bird guided to a bees' nest, the guiding bird was the first bird to arrive at the beeswax at nine sites (39%). We tested whether this value differed from chance. We estimated the number of honeyguides present at 30 harvest sites to calculate how frequently we would expect the guiding bird to arrive at the beeswax first, if its odds of doing so were the same as those of other birds present. Our results strongly suggested that the observed frequency of the guiding bird arriving at the beeswax first were significantly greater than those expected under random chance (figure 3, permutation test *p* < 0.001, mean expected probability 5.6 ± 23%). As such, we estimate the finder's advantage to be a sevenfold increase in the likelihood of arriving at the beeswax first. We assessed how sensitive this result is to variation in our estimate of the number of honeyguides present. This sensitivity analysis indicated that even if we re-generated the permutations using the lower bound of the 95% confidence interval of our estimate of the number of honeyguides density, the observed frequency of the guiding bird arriving first remained significantly greater than the expected frequency (see electronic supplementary materials).

## 4. Discussion

We studied the consistency, correlates and consequences of foraging tactics in a wild, naturally occurring producer–scrounger system in which producer honeyguides cooperate with humans to locate and access bees' nests. Our analyses revealed, first, that guiding (the producer tactic) and scrounging of beeswax show weak individual repeatability, with honeyguides readily switching between tactics. Second, the propensity to guide was predicted by skeletal body size: larger honeyguides (as measured by their tarsus length) guided less and scrounged more. In males, body mass was unrelated to guiding or scrounging behaviour, while in females, the heaviest individuals were substantially more likely to guide than the lightest individuals. Contrary to the hypothesis that higher quality individuals should scrounge more and produce less [17], guiding propensity was independent of our markers of condition (relative telomere length and plumage quality). Third, guiding yielded a significant finder's advantage, upholding a core assumption of many theoretical models of producer–scrounger dynamics.

We found that honeyguides readily switched between guiding and scrounging for beeswax. This finding aligns with previous work suggesting that individual tactic specialization should be rare in producer–scrounger systems, and occur only where genetic factors constrain tactics or where phenotypic differences strongly determine the pay-offs ([7] and references therein; see also [54]). Though some phenotypic traits were significantly related to choice of tactic in our population (see below), this pattern appears not to be strong enough to generate individual specialization. Instead, we suggest that tactic flexibility is likely to be adaptive in honeyguides, for two reasons. First, finding a willing honey-hunter who goes on to harvest a bees' nest is likely to be unpredictable, which will limit the long-term rewards of a pure producer strategy. Second, when beeswax becomes available, it is locally abundant because (at least in our study population) the beeswax reward is generous and provides extensive scrounging opportunities [32]. As such, even for individuals capable of guiding, it would pay to opportunistically scrounge.

Our results provide some support for the hypothesis that phenotypically higher quality individuals should scrounge rather than produce, because larger honeyguides (as measured by their tarsus length) were less likely to guide. Work on captive house sparrows (*Passer domesticus*) indicates that more dominant individuals can increase the pay-offs of scrounging by competitively displacing rivals at the food resource [11,12]. As such, honeyguides with longer tarsi may be able to reliably feed on beeswax without incurring the energetic costs and predation risks likely to be associated with locating a willing honey-hunter and conspicuously signalling while guiding them. Scrounging therefore allows stronger individuals to out-source the costs of food acquisition to producers [55].

Two results were not consistent with the predicted link between scrounging and high individual quality. First, our condition indices were not associated with the tactic an individual adopted. Fault bars can cause feather breakages that impair flight performance [40], and relative telomere lengths reflect lifelong somatic state and predict foraging strategies in other species [56,57], yet neither condition measure was associated with producing or scrounging in honeyguides. Second, the lightest females (those that should be least competitive) never guided and always scrounged. This sex-specific negative association between mass and guiding may be explained by a unique aspect of honeyguides’ biology. In this brood-parasitic species, host choice is inherited maternally, giving rise to two ancient and genetically distinct matrilines specializing on different groups of hosts [58]. Birds in the ‘ground-nesting’ lineage are significantly lighter than those in the ‘tree-nesting’ lineage [58], and so probably comprise the majority of the lighter females in our dataset. Our study was conducted during the breeding season, when ground-nesting females should be focused on searching for terrestrial burrows of their hosts (primarily *Merops* species). This may, first, feasibly reduce their ability to monitor bees' nests in tree cavities. Second, these hosts have a shorter breeding season than some tree-nesting hosts, with peak egg-laying occurring in September and October [59] when our data collection took place. Thus, females from this lineage may have been more focused on reproduction regardless of their knowledge of bees’ nests. Genetic analyses are required to fully explore potential lineage-related differences in guiding propensity. Alternatively, conspicuous guiding calls could draw unwanted attention to lighter females, including threats from predators, from males seeking a mate, or from host and other species which may attack brood-parasitic honeyguides [44]. As such, certain individuals may be limited in their ability to produce, either by incompatibilities with other aspects of their lifestyle, or because their phenotypic traits differentially elevate the costs of producing.

Both theoretical and empirical work suggest that a large food source favours scrounging and lowers producer activity by depressing the finder's advantage [7,24,25], yet guiding behaviour thrives at our study site despite sizeable beeswax rewards. While these generous rewards may be intended for the guiding bird, they more likely feed many additional scroungers than incentivize the guiding bird to guide again. Our finding that the guiding bird is seven times more likely to arrive first at the beeswax helps explain how guiding persists despite the large food source, because although the beeswax reward is large, a smaller portion of newly produced, empty white wax comb is particularly valuable to honeyguides [32,44,45]. The guiding bird is therefore more likely to have the opportunity to feed on this preferred white wax comb. Furthermore, previous work has shown that the initially abundant beeswax reward can be rapidly depleted by an ecological guild of wax-scavengers including honey badgers (*Mellivora capensis*) and African civets (*Civettictis civetta*) [32]. As a result, scroungers have limited access to the best elements of the food source and risk being deprived altogether, while the producer is typically guaranteed a feeding opportunity (and often on the highest value parts of the resource). Theoretical work has considered quality differences *among* food patches [7], but to our knowledge, resources composed of a mix of high- and low-quality items have not been considered. Heterogeneity within food patches is likely to be common in nature and could strongly affect the finder's advantage, as the early-arriving individual is expected to monopolize the highest-quality elements of the resource.

Our results suggest that human–honeyguide cooperation is not jeopardized by cheating behaviour in which individuals scrounge beeswax rather than cooperating with humans. The substantial finder's advantage ensures that any individual capable of guiding should be incentivized to guide (rather than scrounge). Moreover, extensive tactic-switching results in an entire population of potential guides (rather than a restricted minority of consistent producers). As such, a honey-hunter wishing to increase their honey harvest by cooperatively engaging with a honeyguide should be able to relatively quickly find an individual willing and able to adopt the producer tactic, despite prevalent scrounging behaviour. Human–honeyguide cooperation is similarly robust to exploitation by heterospecific wax-eating species [32], and our results are in-line with studies indicating that several other mutualisms are similarly robust to exploitation or cheating. For example, nectar-robbing insects do not disrupt plant-pollinator mutualisms [60] and cleaning mutualisms are not threatened by cleaner-fish that eat their client's tissue rather than removing ecto-parasites [61] (but see [62]). As such, our results add to growing evidence that the threat exploiter or scrounger individuals pose to the persistence of mutualisms appears to be substantially smaller than previously suggested [63,64].

How the intensity of the producer–scrounger game (i.e. the number of opportunities to scrounge) affects the robustness and productivity of the human–honeyguide mutualism is less clear. Opportunities to scrounge appear to be driven primarily by the beeswax reward traditions of a given honey-hunting culture. Across Africa, these rewards range from large excesses (e.g. up to 1.5 kg beeswax for the Yao community in the current study [32]) to extremely limited rewards (e.g. the Hadzabe and Awer honey-hunters in Tanzania and Kenya, respectively [29,31,33]). As such, the producer–scrounger game remains productive despite substantial variation in the opportunities to scrounge.

Both ecological and behavioural processes likely help to keep the human–honeyguide mutualism productive regardless of such variation in scrounging opportunities. We suggest that beeswax scarcity (as in the Hadzabe or Awer reward traditions) promotes the producer tactic by limiting opportunities to scrounge and increasing the finder's advantage [5,32]. Conversely, beeswax abundance (as in the Yao reward tradition) may maintain the producer tactic through three non-mutually exclusive processes. First, as noted above, larger rewards may in fact feed a limited number of scroungers, because the beeswax is soon depleted by mammalian beeswax scavengers [32]. Second, larger rewards could sustain a higher density of honeyguides, all of which are able to guide in the future due to tactic-switching. Finally, larger rewards may give naïve individuals opportunities to socially learn how better to guide honey-hunters, reinforced by a scrounged beeswax meal. Indeed, in other systems, tactic choice depends on prior experience and scrounging allows individuals to socially learn how to produce [36–39,65]. Although we did not find that younger honeyguides scrounged more often, our dataset contains only individuals over approximately 1 year old and further work is needed to investigate whether guiding skills are honed while scrounging during the first year of life. In the honeyguide system, these potential learning opportunities are determined by human cultural traditions dictating the size of beeswax rewards. Thus, socially learnt human behaviour may reciprocally influence opportunities for social learning in honeyguides, and help maintain a productive mutualism regardless of the intensity of the producer–scrounger game that underpins it.

This study also has broader implications for our understanding of the future of human–wildlife cooperation [27,34], which may depend on the number of individuals willing and able to engage with human partners. Our analyses suggested that, in honeyguides, all individuals are capable of cooperating with humans, consistent with the hypothesis that a tendency to guide humans is innate in honeyguides [27,30]. By contrast, in both human–dolphin (*Tursiops truncatus gephyreus*) and the now-extinct human–orca (*Orcinus orca*) cooperation, only a small subset of individuals help(ed) humans catch fish and whales, respectively, and this ability is culturally inherited [66,67]. These factors appear to have contributed to the loss of human–orca cooperation, when one of the most cooperative individuals was accidently killed [67], and the declining human–dolphin cooperative partnership in Brazil faces a similar risk [68]. Indeed, human–dolphin cooperation has already ceased at other locations [69]. The death of a single cooperative dolphin would likely represent a substantial loss for local fishermen [70], while the death of a single guiding honeyguide would be extremely unlikely to affect local honey-hunters. Our results are consistent with the hypothesis that a widespread, apparently innate willingness to cooperate with humans makes human–honeyguide cooperation less likely to decline or go extinct altogether [34].

## 5. Conclusion

Our findings suggest that the human–honeyguide mutualism is robust against scrounging, because tactic-switching and a sizeable finder's advantage ensure that most honeyguides should be able and willing to cooperate with a honey-hunter when the opportunity arises. Broadly, our results suggest that even in systems in which switching between producing and scrounging is prevalent, phenotypic traits related to competitive ability can predispose certain individuals to one tactic, likely by moderating its costs and benefits. These trait-related differences in foraging strategy may have implications for the function and resilience of producer–scrounger systems. Finally, considering the heterogeneous nature of food resources reveals insights into the finder's advantage gained by early-arriving producers which would otherwise be cryptic.

## Data Availability

Supplementary material is available online [71].
